# Clinicopathological Study of Soft Tissue Neoplasms

**DOI:** 10.7759/cureus.83576

**Published:** 2025-05-06

**Authors:** Anuja U Paul, Shrikant M Mane, Sunil Y Swami, Alka Gore

**Affiliations:** 1 Pathology, Bharati Vidyapeeth (Deemed to be University) Medical College and Hospital, Sangli, Sangli, IND; 2 Pediatric Cardiology, Bharati Vidyapeeth (Deemed to be University) Medical College and Hospital, Sangli, Sangli, IND; 3 Pathology, Vilasrao Deshmukh Government Medical College, Latur, Latur, IND; 4 Biostatistics, Bharati Vidyapeeth (Deemed to be University) Medical College and Hospital, Sangli, Sangli, IND

**Keywords:** anatomical site distribution, benign soft tissue tumors, clinicopathological study, histopathology, incidence, malignant soft tissue tumors, relative frequency, sarcoma, sex ratio, soft tissue neoplasms

## Abstract

Background

Soft tissue neoplasm is a broad term referring to tumors that can arise from various tissues, including muscle, tendons, fat, nerves, and lymphatic and blood vessels. These neoplasms may be either benign or malignant, with classification based on the tissue of origin and tumor behavior. The aim of this study was to assess the relative frequency of soft tissue neoplasms among all neoplasms, determine the incidence of benign and malignant soft tissue tumors (STTs), and examine variations in age, sex, anatomical location, and histological subtypes.

Methods

This descriptive study was conducted at Swami Ramanand Teerth Rural Government Medical College, Ambajogai, India, from November 2015 to November 2017. It included cases of soft tissue neoplasms diagnosed through clinical history and examination, followed by biopsy or surgical excision and histopathological analysis.

Results

A total of 200 soft tissue neoplasm cases were identified out of 4,494 neoplasms recorded during the study period, accounting for 4.45% of all neoplasms. Of these, 188 cases (94%) were benign and 12 cases (6%) were malignant. Benign neoplasms were most common in individuals aged 21-40 years and showed a slight male predominance. The upper extremity was the most frequent site for benign STTs, followed by the trunk, head and neck, and lower extremity. In contrast, malignant soft tissue neoplasms were most commonly found in the lower extremity. Among the 200 soft tissue neoplasms, adipocytic tumors were the most prevalent, comprising 105 cases (52.5%), followed by vascular neoplasms with 32 cases (16%) and peripheral nerve sheath tumors with 22 cases (11%).

Conclusions

This study provides insight into the clinicopathological features of soft tissue neoplasms, highlighting their distribution across different age groups, sexes, anatomical sites, and histological subtypes.

## Introduction

Soft tissue refers to all non-epithelial, extra-skeletal tissues in the body that are not part of the reticuloendothelial system. This category includes glial tissue and the supportive connective tissues of various parenchymal organs. Voluntary muscles, adipose tissue, fibrous tissue, and their associated blood vessels are all considered soft tissue. Conventionally, the peripheral nervous system is also included, as tumors originating from nerves often present as soft tissue masses and pose similar challenges in differential diagnosis and treatment [1].

Embryologically, most soft tissues derive from the mesoderm, with some contribution from the neuroectoderm [1]. Neoplasms of mesenchymal origin occur far less frequently than those of ectodermal or endodermal origin [2]. Soft tissue tumors (STTs) are classified into benign, intermediate, and malignant categories based on their biological behavior [3]. Intermediate tumors are further subdivided into those that are locally aggressive and those that rarely metastasize.

Unlike carcinomas and other common neoplasms, soft tissue sarcomas are rare, accounting for less than 1% of all cancers [4]. Benign STTs are more common than their malignant counterparts [5].

A correlation is observed among tumor type, symptoms, anatomical location, patient age, and sex. Lipomas are typically asymptomatic, uncommon in areas such as the hands, lower legs, and feet, and are exceedingly rare in children. Malignant STTs can appear in various anatomical sites, with 75% occurring in the extremities, especially the thigh, followed by 10% in the trunk wall and another 10% in the retroperitoneum. A slight male predominance is noted [3]. Benign STTs are most frequently diagnosed in individuals in their second to fourth decades of life, while malignant tumors are more often seen in older patients. An exception is embryonal rhabdomyosarcoma (RMS), which has been observed in younger individuals [6].

The majority of STTs are benign and have a high cure rate with surgical excision. However, malignant mesenchymal tumors can be life-threatening and are diagnostically and therapeutically challenging, primarily due to the existence of more than 50 histological subtypes of soft tissue sarcomas [7].

Clinical evaluation, including patient age, duration of symptoms, tumor location and size, imaging findings, and histopathological examination, remains the most reliable method for accurate diagnosis and for predicting tumor behavior [5]. Fine-needle aspiration cytology (FNAC) also plays an important diagnostic role, particularly for superficial lesions [8]. CT-guided FNAC is useful for identifying intra-abdominal and retroperitoneal tumors, although its sensitivity and specificity are subjects of ongoing debate due to known limitations [8]. As a result, histopathological examination is widely regarded as the gold standard for diagnosing soft tissue malignancies. Diagnostic accuracy can be further improved using special techniques such as special stains, immunohistochemistry (IHC), electron microscopy, and cytogenetic or molecular studies [9].

The aim of this study was to determine the relative frequency of soft tissue neoplasms among all neoplasms and to evaluate the incidence of both benign and malignant STTs in the hospital population over a two-year period. Additionally, the study sought to analyze differences in tumor presentation based on age, sex, anatomical location, and histological features, while also comparing these findings with those reported in earlier studies. Thus, this research was undertaken with the novel aim of exploring the clinicopathological characteristics of soft tissue neoplasms.

## Materials and methods

This descriptive study was conducted at the Department of Pathology, Swami Ramanand Teerth Rural Government Medical College, Ambajogai, India, over a two-year period from November 2015 to November 2017. During this time, a total of 4,494 neoplasm cases were documented, of which 200 were identified as soft tissue neoplasms.

All excisional biopsy specimens submitted to the pathology department for histopathological evaluation during the study period were thoroughly examined through clinical, radiological, and histological assessments. Only cases confirmed as soft tissue neoplasms were included in the study. Exclusion criteria comprised patients who received conservative treatment, those referred to other healthcare facilities, cases diagnosed through incisional biopsy, and STTs arising in systemic organs, such as uterine leiomyoma.

The study was approved by the Institutional Ethics Committee of Swami Ramanand Teerth Rural Government Medical College, Ambajogai (approval number SRTRGMC/phar/IEC/233/15).

This retrospective descriptive analysis focused on 200 confirmed cases of soft tissue neoplasms. Clinical data, including age, sex, anatomical site, clinical diagnosis, relevant investigations, and histological findings, were retrieved from the histopathology records. Anatomical locations were categorized as follows: upper extremity (shoulder, arm, forearm, wrist, and hand), lower extremity (buttock, thigh, leg, and foot), trunk (back and chest wall), head and neck, and miscellaneous regions.

All specimens were preserved in 10% formalin. After fixation, gross features such as size, morphology, color, and texture were recorded. Representative sections measuring approximately 1 × 1.5 cm and 4 mm in thickness were taken, and images were captured for select specimens.

Each specimen was assigned a unique identification number and placed into a tissue cassette for processing. An automated tissue processor carried out a 12-step cycle over approximately 18 hours, preparing the samples through fixation, dehydration, clearing, and paraffin infiltration. Paraffin-embedded sections, 3-5 μm thick, were prepared using a rotary microtome. These sections were then stained with H&E, with special stains used as necessary. The slides were mounted using DPX to create permanent preparations.

Microscopic evaluation was performed using a light microscope. Clinical, radiological, gross, and histological findings were correlated, and challenging cases were reviewed with input from expert and senior pathologists to establish a final diagnosis.

Histological subtypes of STTs were classified according to the 2020 WHO classification of STTs [10]. Data analysis was conducted using Microsoft 365 (Microsoft Corporation, Redmond, WA, USA) and IBM SPSS Statistics for Windows, Version 22.0 (Released 2013; IBM Corp., Armonk, NY, USA). Results were tabulated, with categorical variables presented as percentages and continuous variables expressed as SDs.

## Results

Over the two-year study period, the pathology department evaluated a total of 4,494 neoplasms. Of these, 200 cases (4.45%) were identified as soft tissue neoplasms, comprising 188 benign tumors (4.18%) and 12 malignant tumors (0.27%) (Table 1).

**Table 1:** Comparison of the frequency of occurrence between benign and malignant soft tissue neoplasms

Type	Benign (%)	Malignant (%)	Total number (%)
All neoplasms	4,187 (93.17%)	307 (6.83%)	4,494 (100%)
Soft tissue neoplasms	188 (4.18%)	12 (0.27%)	200 (4.45%)

Benign soft tissue neoplasms were observed in patients ranging from two to 80 years of age, with the highest incidence occurring in the 21- to 40-year age group. Malignant soft tissue neoplasms were found in patients aged two to 75 years, most commonly presenting in the fifth to seventh decades of life (Table 2).

**Table 2:** Age-wise distribution (in years) of patients with soft tissue neoplasms B: benign, M: malignant; PNST: peripheral nerve sheath tumor

Serial number	Neoplasms	0-20		21-40		41-60		61-80	
		B	M	B	M	B	M	B	M
1	Adipocytic	4	0	41	0	44	1	15	0
2	Fibroblastic	2	0	7	0	6	1	0	2
3	Fibrohistiocytic	2	0	4	0	4	0	0	0
4	Vascular	4	0	18	0	7	0	3	0
5	Smooth and skeletal muscle	0	1	1	0	2	1	1	4
6	PNST	5	0	8	0	6	0	3	0
7	Uncertain differentiation	1	1	0	1	0	0	0	0
8	Total	18 (9.6%)	2 (16.6%)	79 (42.0%)	1 (8.3%)	69 (36.7%)	3 (25%)	22 (11.7%)	6 (50%)

Among benign soft tissue neoplasms, 109 cases (58%) occurred in males and 79 cases (42%) in females, yielding a male-to-female ratio of 1.4:1. In malignant soft tissue neoplasms, eight cases (66.6%) were seen in males and four cases (33.3%) in females, resulting in a male-to-female ratio of 2:1 (Table 3).

**Table 3:** Sex-wise distribution of soft tissue neoplasms PNST: peripheral nerve sheath tumor

Serial number	Neoplasms	Benign		Malignant	
		Male (%)	Female (%)	Male (%)	Female (%)
1	Adipocytic	65 (34.6%)	39 (20.7%)	1 (8.33%)	0 (0%)
2	Fibroblastic	4 (2.2%)	11 (5.9%)	2 (16.66%)	1 (8.33%)
3	Fibrohistiocytic	7 (3.7%)	3 (1.6%)	0 (0%)	0 (0%)
4	Vascular	19 (10.1%)	13 (6.9%)	0 (0%)	0 (0%)
5	Smooth and skeletal muscle	4 (2.1%)	0 (0%)	4 (33.33%)	2 (16.66%)
6	PNST	9 (4.8%)	13 (6.9%)	0 (0%)	0 (0%)
7	Uncertain differentiation	1 (0.5%)	0	1 (8.33%)	1 (8.33%)
8	Total	109 (58%)	79 (42%)	8 (66.6%)	4 (33.3%)

Benign soft tissue neoplasms were distributed across various anatomical regions, with the highest number of cases occurring in the upper extremities (54 cases, 28.7%), followed by the trunk (47 cases, 25%), head and neck region (44 cases, 23.4%), and lower extremities (32 cases, 17%). In contrast, malignant soft tissue neoplasms were most commonly located in the lower extremities, which accounted for five cases (41.7%) (Table 4).

**Table 4:** Anatomical site-wise distribution of soft tissue neoplasms B: benign, M: malignant; PNST: peripheral nerve sheath tumor

Serial number	Neoplasms	Upper extremity		Lower extremity		Trunk		Head and neck		Other	
		B	M	B	M	B	M	B	M	B	M
1	Adipocytic	20	1	20	0	38	0	20	0	6	0
2	Fibroblastic	8	0	2	1	2	0	0	1	3	1
3	Fibrohistiocytic	8	0	1	0	1	0	0	0	0	0
4	Vascular	9	0	2	0	1	0	19	0	1	0
5	Smooth and skeletal muscle	2	0	0	3	1	1	0	1	1	1
6	PNST	7	0	6	0	4	0	5	0	0	0
7	Uncertain differentiation	0	0	1	1	0	0	0	0	0	1
8	Total	54 (28.7%)	1 (8.3%)	32 (17.0%)	5 (41.7%)	47 (25.0%)	1 (8.3%)	44 (23.4%)	2 (16.6%)	11 (5.9%)	3 (25%)

**Figure 1:**
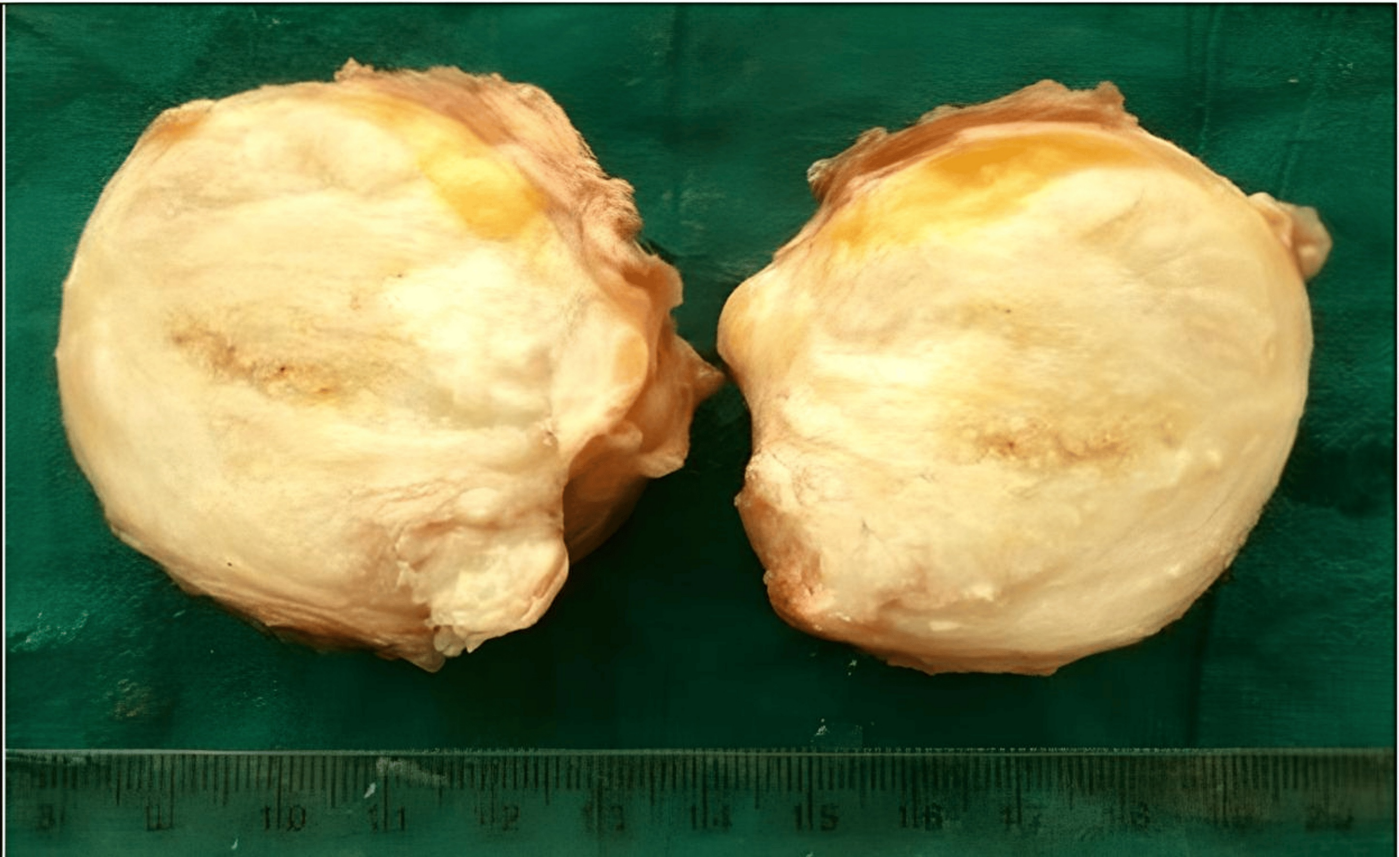
Spindle cell lipoma: a well-circumscribed mass with a gray-white cut surface

**Figure 2:**
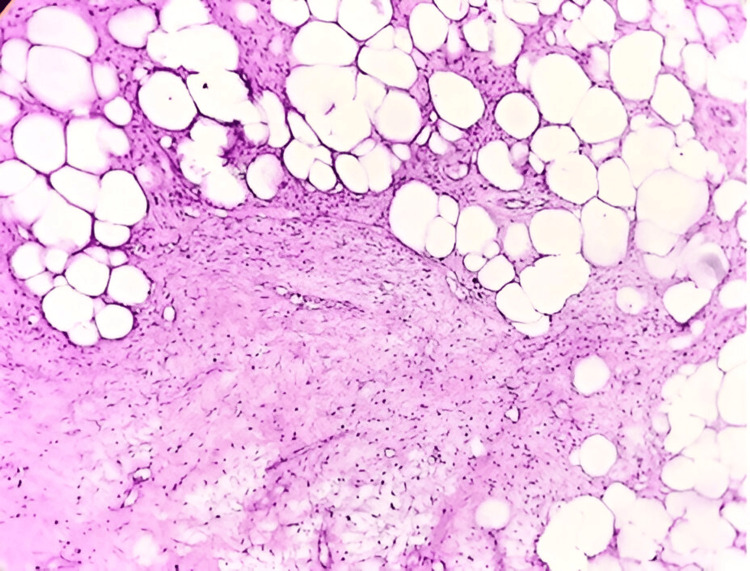
Spindle cell lipoma: displaying an admixture of mature fat cells and spindle cells, along with a fine vascular network, mast cells, and myxoid changes (H&E: 10X)

Benign adipocytic tumors comprised 104 cases (52%) of all soft tissue neoplasms, with lipoma being the most common subtype, accounting for 90 cases (47.9%). Other variants included fibrolipoma (seven cases, 3.7%), spindle cell lipoma (two cases, 1.1%), nevus lipomatosus superficialis (two cases, 1.1%) (Figure 1, Figure 2), and single cases of myxolipoma, intramuscular lipoma, and pleomorphic lipoma. These tumors were observed across all age groups (two to 80 years), with a male-to-female ratio of 1.7:1. The trunk was the most frequently affected anatomical site.

**Figure 3:**
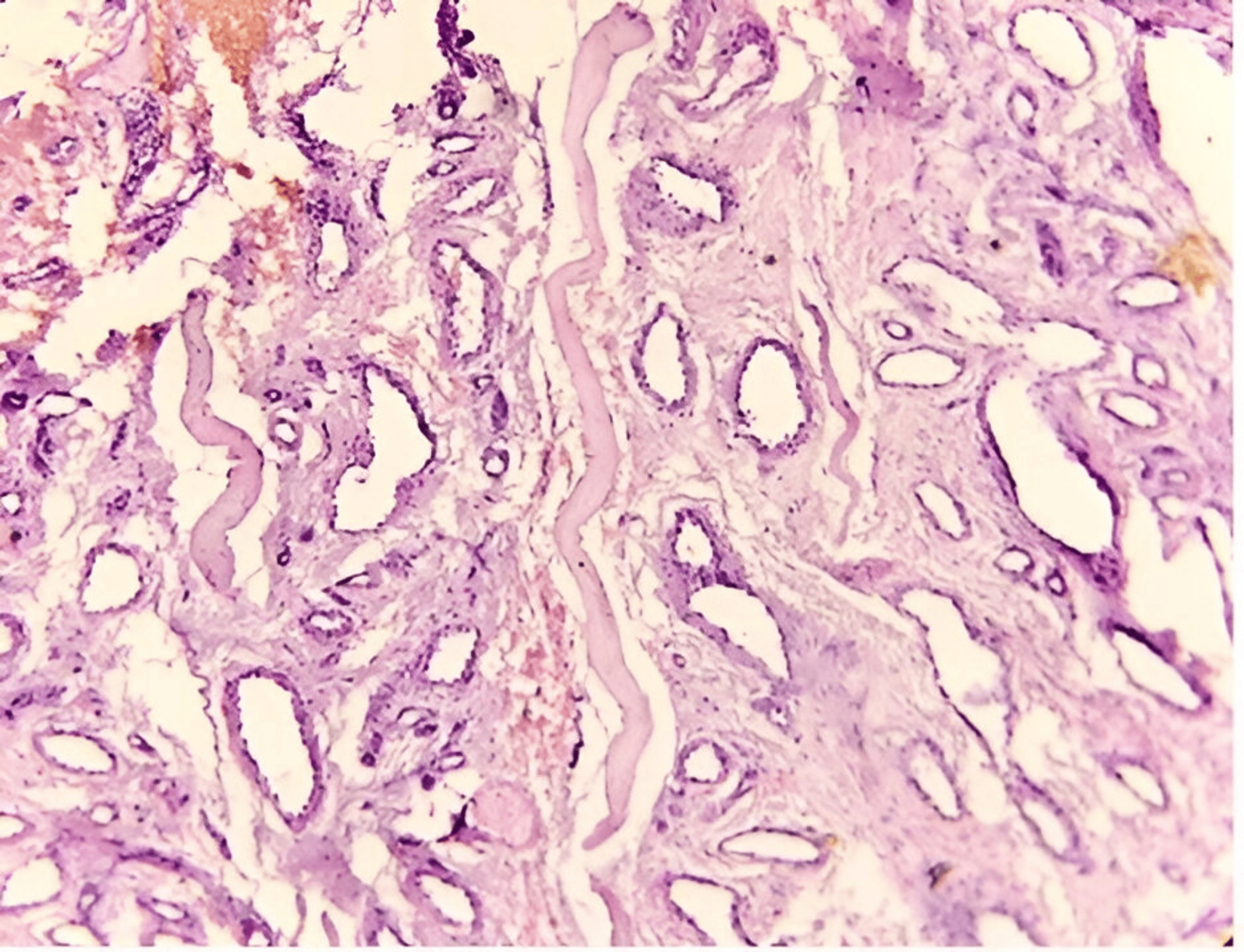
Intramuscular hemangioma: showing separation of muscle fibers with numerous proliferating blood vessels (H&E: 10X)

Benign vascular tumors (Figure 3) were the second most common tumor category, comprising 32 cases (16%). These predominantly involved the head and neck region and exhibited a male-to-female ratio of 1.5:1 (Table 5).

**Table 5:** Benign soft tissue neoplasms: distribution according to histological subtypes, frequency, age, sex, and common site

Serial number	Histological type	Cases (%)	Age (years)	Sex (M/F)	Common site
1	Lipoma	90 (47.9%)	12-80	58M/32F	Trunk
2	Nevus lipomatosus superficialis	2 (1.1%)	60-65	1M/1F	Lower extremity and head/neck
3	Fibrolipoma	7 (3.7%)	2-50	3M/4F	Lower extremity
4	Myxolipoma	1 (0.5%)	25	0M/1F	Lower extremity
5	Intramuscular lipoma	1 (0.5%)	50	1M/0F	Head/Neck
6	Spindle cell lipoma	2 (1.1%)	45-60	2M/0F	Upper extremity, head/neck
7	Pleomorphic lipoma	1 (0.5%)	36	0M/1F	Upper extremity
8	Fibroma	6 (3.2%)	23-60	1M/5F	Upper extremity
9	Desmoid tumor	4 (2.1%)	25-50	4F	Abdomen
10	Superficial acral fibromyxoma	2 (1.1%)	15-55	2M	Upper extremity
11	Myositis ossificans	1 (0.5%)	12	1F	Lower extremity
12	Nodular fasciitis	1 (0.5%)	60	1M	Upper extremity
13	Elastofibroma	1 (0.5%)	58	1F	Trunk
14	Giant cell tumor of the tendon sheath	7 (3.7%)	8-60	5M/2F	Upper extremity
15	Benign fibrous histiocytoma	3 (1.6%)	45-58	2M/1F	Upper extremity, trunk
16	Hemangioma	14 (7.5%)	8-65	9M/5F	Head/neck, upper extremity
17	Intramuscular hemangioma	1 (0.5%)	21	1F	Trunk
18	Pyogenic granuloma	17 (9.0%)	21-65	10M/7F	Head/neck
19	Angioleiomyoma	3 (1.6%)	40-60	3M	Trunk, upper extremity
20	Cutaneous leiomyoma	1 (0.5%)	70	1M	Scrotum
21	Neurofibroma	11 (5.9%)	6-80	4M/7F	Upper extremity
22	Schwannoma	10 (5.3%)	15-65	5M/5F	Lower extremity, trunk
23	Extraneural perineurioma	1 (0.5%)	60	1F	Lower extremity
24	Xanthoma	1 (0.5%)	39	1M	Lower extremity

Benign peripheral nerve sheath tumors (PNSTs) were the third most common, accounting for 22 cases (11%). These tumors occurred in patients aged six to 80 years and were mainly located in the extremities, with a slight female predominance (male-to-female ratio of 1:1.4). Neurofibromas and schwannomas were the most frequently identified subtypes in this group.

**Figure 4:**
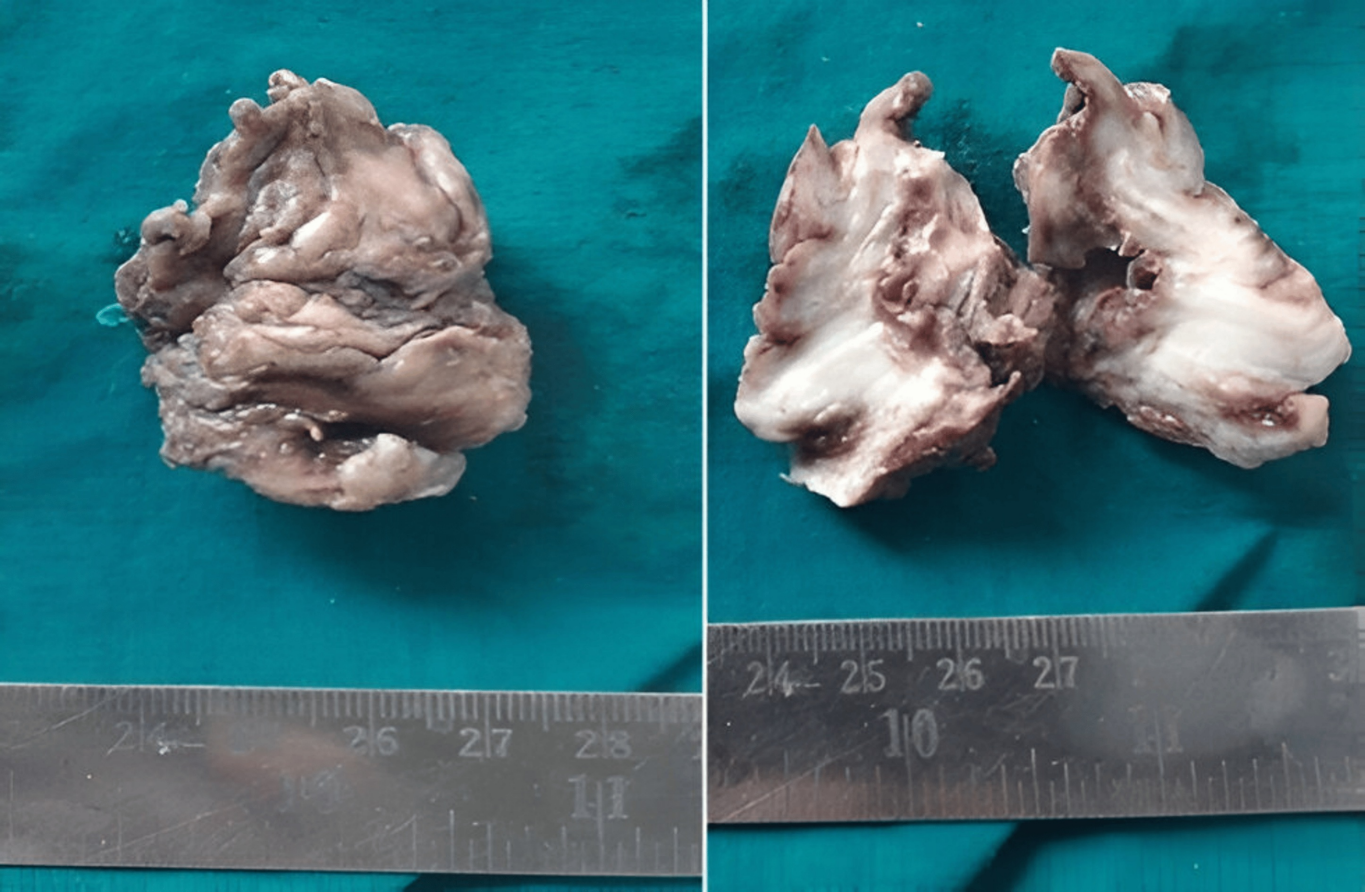
Elastofibroma: an irregular, firm tissue mass with a gray-white cut surface

**Figure 5:**
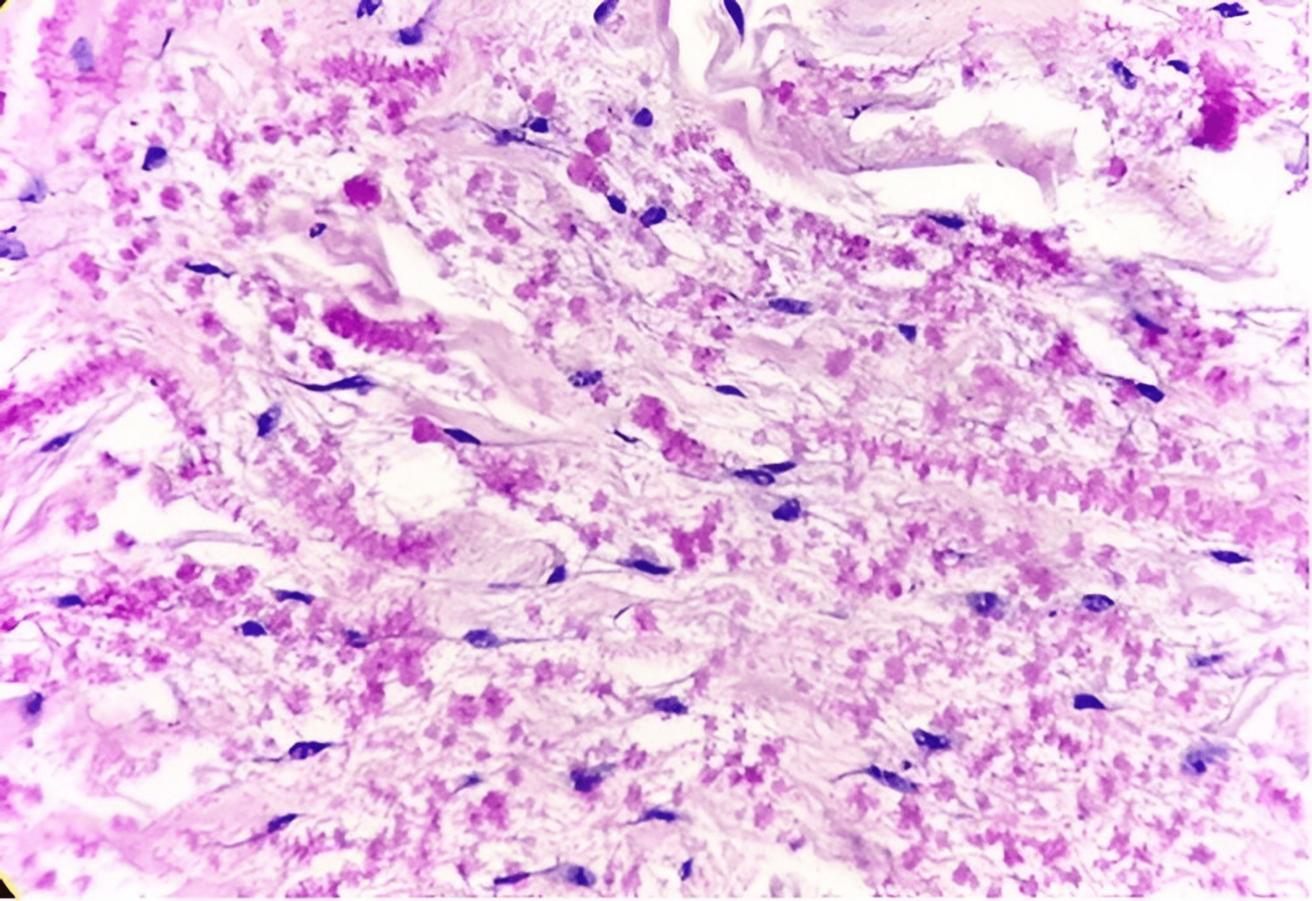
Elastofibroma: demonstrating altered elastic fibers within a collagenous matrix (H&E: 40X)

Benign fibroblastic tumors (Figure 4, Figure 5) accounted for 15 cases (7.5%), seen in individuals aged 12 to 60 years. These tumors showed a strong female predominance (male-to-female ratio of 1:2.7) and most commonly affected the upper extremities.

Benign fibrohistiocytic tumors made up 10 cases (5%) of all benign soft tissue neoplasms, while smooth and skeletal muscle tumors accounted for four cases (2%). A single case of a benign tumor of uncertain differentiation was also identified (Table 5).

**Figure 6:**
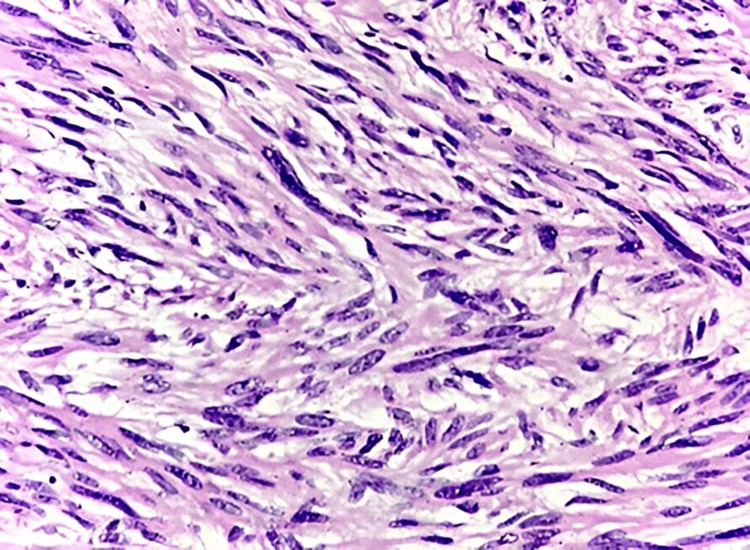
LMS: showing a tumor composed of spindle cells arranged in a fascicular pattern, with cigar-shaped pleomorphic and hyperchromatic nuclei, and cytoplasmic vacuoles at both ends of the nuclei (H&E: 40X) LMS, leiomyosarcoma

In our analysis of malignant soft tissue sarcomas, the most common types were those originating from smooth and skeletal muscle, followed by fibroblastic tumors. Three cases of leiomyosarcoma (LMS) (25%) (Figure 6) were identified in the elderly population, aged 60 to 70 years, with a male-to-female ratio of 1:2. These tumors predominantly affected the extremities. We also identified three cases of pleomorphic RMS (PRMS) (25%) in patients aged two to 72 years, with a male predominance. These tumors were located in the lower extremities, trunk, and head and neck regions.

Malignant fibroblastic tumors were categorized into three subtypes based on their histology: myxoinflammatory fibroblastic sarcoma, low-grade fibromyxoid sarcoma derived from dermatofibrosarcoma protuberans (DFSP), and fibrosarcoma also arising from DFSP. Other soft tissue sarcomas included a single case each of myxoid liposarcoma, angiomatoid fibrous histiocytoma (AFH), and synovial sarcoma (Table 6).

**Table 6:** Malignant soft tissue neoplasm group: distribution according to histological subtypes, frequency, age, sex, and common site DFSP: dermatofibrosarcoma protuberans; LGFMS: low-grade fibromyxoid sarcoma; MIFS: myxoinflammatory fibroblastic sarcoma

Serial number	Histological group	Histological type	Number of cases (n, %)	Age in years	Sex (M/F)	Common site
1	Adipocytic	Myxoid liposarcoma	1 (8.33%)	50	1M	Upper extremity
2	Fibroblastic	LGFMS	1 (8.33%)	42	1M	Abdomen
3	Fibroblastic	Fibrosarcoma in DFSP	1 (8.33%)	65	1M	Head and neck
4	Fibroblastic	MIFS	1 (8.33%)	75	1F	Lower extremity
5	Smooth and skeletal muscle	LMS	3 (25%)	60-70	1M/2F	Lower extremity and abdomen
6	Smooth and skeletal muscle	PRMS	3 (25%)	2-72	3M	Lower extremity, trunk, and head and neck
7	Uncertain differentiation	AFH	1 (8.33%)	20	1M	Lower extremity
8	Uncertain differentiation	Synovial sarcoma	1 (8.33%)	20	1F	Abdomen

## Discussion

This descriptive research includes 200 cases of soft tissue neoplasms, comprising both benign and malignant types. The study aimed to examine hospital-based data on the number and percentage of benign and malignant STTs, as well as differences in their age, gender, location in the body, and histological features. The term “incidence” in this context refers to occurrences within our hospital, not the broader population.

Benign soft tissue neoplasms significantly outnumbered malignant ones across all investigations. Our study identified 188 cases (94%) of benign soft tissue neoplasms and 12 cases (6%) of malignant soft tissue neoplasms. Myhre-Jensen conducted a consecutive seven-year study of 1,331 benign STTs and compared them with data on 72 malignant soft tissue sarcomas diagnosed in the same period [11]. Agravat et al. examined the histology of human STTs and tumor-like lesions and found a benign-to-malignant soft tissue neoplasm ratio of 92:6 [12]. The proportions of benign (94%) and malignant (6%) neoplasms in our study were consistent with the findings of Myhre-Jensen and Agravat et al. [11,12].

Benign adipocytic tumors accounted for 104 cases (52%) of all soft tissue neoplasms, a figure close to that found by Myhre-Jensen and higher than those reported by Agravat et al. and Hasan et al. [13]. In 1995, Kransdorf examined benign soft-tissue tumors in a large referral population with various diagnoses based on age, sex, and location, finding that only 16.1% of adipocytic tumors were present [14]. The proportion of adipocytic tumors in our analysis was significantly higher than in these previous studies, possibly due to intrinsic bias within the referral population. The second most prevalent tumor category was vascular tumors, comprising 32 cases (16%), consistent with Myhre-Jensen’s study. The next most prevalent group was PNST, accounting for 22 cases (11%), matching Hasan et al.’s findings [13].

Ramnani et al. investigated the clinicopathological features of benign STTs [15], finding that lipomas and their variants constituted the largest percentage of cases at 50.8%. Lipomas primarily manifested in individuals aged 21-50 years, with the highest prevalence (37.7%) in the 31- to 40-year age group, showing a male predominance. Our study indicates that lipoma (52%) is the most prevalent STT, with a peak incidence in the third and fourth decades and a male-to-female ratio of 1.7:1. The trunk was the most common site of incidence, consistent with all major study groups. Ninety cases of lipoma were recorded, followed by seven cases of fibrolipoma, and two cases each of spindle cell lipoma and nevus lipomatosus superficialis, predominantly occurring in the third and fourth decades of life with a male predominance. There was one case each of myxolipoma, intramuscular lipoma, and pleomorphic lipoma. Spindle cell lipomas showed a male predominance, consistent with Syed et al.’s findings, who reported 22 males out of 28 cases [16].

In the benign vascular neoplasm group, which comprised 32 cases (16%), the predominant site of involvement was the head and neck region. There were 22 cases (11%) of benign PNST within the six- to 80-year age range, with the extremities being the most common site. Benign fibroblastic neoplasms accounted for 15 cases (7.5%). These findings align with those of Agravat et al. [12], Kransdorf [14], and Myhre-Jensen [11]. The benign fibrohistiocytic neoplasms accounted for 10 cases (5%), lower than other studies due to differences in sample size.

Begum et al. conducted a clinicopathological investigation on soft tissue neoplasms, revealing an age incidence ranging from six to 77 years, with a peak in the fourth decade [5]. In our study, the age incidence of benign soft tissue neoplasms ranged from 2 to 80 years, with a peak in the fourth decade. The average age for malignant soft tissue neoplasms was 51.3 years, consistent with Myhre-Jensen’s study. Both benign and malignant soft tissue neoplasms were slightly more common in men, in line with findings by Kransdorf [14,17] and Athulya and Ariya [18]. Our study agrees with Kransdorf [14], showing that most benign soft tissue neoplasms (54 cases, 28.7%) were found in the upper extremities. We identified a maximum of six (50%) malignant soft tissue neoplasms in the extremities. Venkatraman et al. [19] also observed that 75% of their cases manifested in the extremities. Aliyu et al. [20], Jenna et al. [21], and Gulia et al. [22] noted an increased propensity for these tumors in the extremities as well.

In this study, the most common types of malignant soft tissue sarcomas were malignant fibroblastic tumors, smooth muscle tumors, and skeletal muscle tumors, each accounting for three cases (25% of the total). We identified one case each of synovial sarcoma, myxoid liposarcoma, and AFH, each representing 8.33%. The incidence of synovial sarcoma was similar to that reported by Kransdorf [17]. The three cases of fibrosarcoma (25%) exceeded findings by Kransdorf and Agravat et al., likely due to differences in sample size. Three cases of LMS and three cases of RMS, each accounting for 25%, also surpassed Kransdorf’s findings. Two cases of tumors of uncertain differentiation were observed in a 20-year-old patient, with a male-to-female ratio of 1:1. The age of occurrence and male-to-female ratio were comparable to those in Kransdorf’s study [17].

Limitations

We acknowledge some limitations in the study. When the histological differentiation is unclear, the histomorphologic pattern observed may assist in determining differential diagnoses, guiding further workup. Due to economic constraints, we were unable to conduct ancillary testing, such as IHC, which plays a crucial role in diagnosing cases of STTs.

## Conclusions

STTs have long intrigued pathologists due to the wide variety of tumor types and the subtle histopathological similarities and differences that can only be detected through careful microscopic examination. Through this study, we were able to reassess the clinicopathological profile of soft tissue neoplasms and their various types with regard to age, sex, and anatomical site distribution.
